# Clinical Impact of Using 4% Icodextrin as an Adhesion Prophylactic Agent in High-Risk Gynecological Laparoscopic Surgery on Hospital Readmission and Reoperation Rates: A Retrospective Single-Arm Study

**DOI:** 10.3390/jcm15083027

**Published:** 2026-04-15

**Authors:** Maya Sophie de Wilde, Kaylen Silverberg, Thamer Alahmad, Rajesh Devassy, Rudy Leon De Wilde, Luz Angela Torres-de la Roche

**Affiliations:** 1 University Hospital for Gynecology, Pius-Hospital Oldenburg, University Medicine Oldenburg, 26121 Oldenburg, Germany; 2 Texas Fertility Center, Austin, TX 78731, USA

**Keywords:** icodextrin, adhesions, readmissions, reoperations, gynecological laparoscopic surgery, adhesion prophylactic agent

## Abstract

**Background/Objectives:** The objective of this study was to evaluate, for the first time, the effectiveness and safety of 4% icodextrin solution by detecting the incidence of adhesion-related morbidities (adhesion-related hospital readmissions, including reoperations) when used as an adhesion prophylactic agent during laparoscopic gynecologic surgery. **Methods:** The study was a single-arm, two-center, retrospective study. The incidence of hospital readmissions that were directly or possibly related to adhesions following the use of 4% icodextrin in laparoscopic gynecologic surgery, 2 years from the date of index surgery, was assessed either via chart review alone or, when found necessary, in combination with patient-completed questionnaires. Patient safety was evaluated through reported adverse events. The relationship between clinical events and the use of 4% icodextrin was assessed by investigators based on patient-level data. **Results:** After 149 patients were screened, the study finally included 123 patients; 4 (3.3%; 95% CI: 0.89%, 8.12%) had at least one reoperation or readmission that was directly or possibly related to adhesion within 2 years of index surgery. In the supplemental analysis (67 patients using chart and questionnaire data), this incidence rate was 10.4% (95% CI: 4.30%, 20.35%). No adverse events related to the use of 4% icodextrin were reported. **Conclusions:** This is the first study ever evaluating hospital readmission/reoperation rates after application of a specific adhesion prophylactic agent. The results indicate that 4% icodextrin is safe and effective when used as an intraperitoneal instillate for reduction in adhesions in gynecological laparoscopic procedures. It has a lower readmission and reoperation rate compared to meta-analysis data in the international literature.

## 1. Introduction

Postoperative adhesions are a significant problem and develop in most patients (55% to 93%) after abdominal or pelvic surgery, often resulting in significant morbidity and complications, such as ileus, subfertility, chronic pelvic pain and small bowel obstruction, which can considerably increase healthcare costs [1,2,3,4,5,6,7]. Adhesion-related reoperations are often more difficult and can lead to additional complications [8], such as increased surgical time, incidence of organ injury and inadvertent enterotomy during reoperations [9].

Good surgical techniques, including a minimally invasive approach, may reduce adhesions and minimize the abovementioned complications. The use of additional methods or agents (solid patches or films, liquids, or gels) is at present indicated according to specific or increased procedural or patient risk [10]. These agents are placed inside the abdominal cavity, especially in the pelvic cavity, during or before completing the surgery, physically preventing unintended contact of traumatized tissues with viscera or peritoneum, as well as reducing the extent and severity of off-site adhesions during the 3- to 5-day period of peritoneal re-mesothelialization [1].

4% Icodextrin Adhesion Reduction Solution (ADEPT ^TM^ Baxter Health Care Corporation, Deerfield, IL, USA) is intended as an intraperitoneal instillate for reducing adhesions following gynecological laparoscopic surgeries in the abdominal pelvic cavity in adults. It is similar in structure to physiologically occurring carbohydrates and is a substrate for amylase, which, although not present in the peritoneal cavity, is present in abundance in the systemic circulation. Icodextrin achieves its function through a physical effect by maintaining a fluid reservoir and thus providing a temporary separation of peritoneal and serosal surfaces by hydroflotation. When administered intraperitoneally as a 1 L 4% solution, icodextrin functions as a colloid osmotic agent, allowing for the retention of a reservoir of fluid within the peritoneal cavity for 3–4 days. This minimizes tissue apposition during the critical period of fibrin formation and mesothelial regeneration following surgery, thereby providing a barrier to adhesion formation, by hydroflotation and dilution of potential adhesion-promoting molecules [11].

The rationale for the current study is to provide additional clinical evidence supporting the safety and performance of 4% icodextrin as an adjunct to good surgical technique for the reduction in post-surgical adhesions in patients undergoing gynecological laparoscopic surgery. To this end, an analysis was conducted on the medical records of patients who had been administered this prophylactic agent following a gynecological procedure.

This is the first study ever reported evaluating the clinical readmission and reoperation rate after application of a specified adhesion prophylactic agent. Therefore, this study is important, as it describes the clinical outcomes and benefits of a specific medical product in reducing adhesion formation.

## 2. Materials and Methods

This study was a single-arm, retrospective study conducted at two centers specialized in minimally invasive surgery (Texas Fertility Center, Austin, TX, USA, and the University Hospital for Gynecology, Pius-Hospital, Oldenburg, Germany). Patient information was obtained by reviewing medical records and patient questionnaires: the last step aimed to supplement the medical records if information relevant to the study could be missing. Data were collected from adult female patient charts (≥18 years of age at the time of surgery) who had undergone an index laparoscopic gynecologic surgery at least 2 years prior to the start of the study and in whom 4% icodextrin was used to prevent post-surgical adhesions. Patients with reported pregnancy, nursing, or a frank infection in the abdominopelvic cavity at the time of surgery were excluded from the analysis.

Patient charts were reviewed to collect data on readmissions related to adhesions [12], which were defined as:

- Directly related to adhesions (adhesiolysis, nonoperative readmissions for adhesions, and adhesiolysis operations on female reproductive tract);
- Possibly related to adhesions (gynecological operations, abdominal surgery, and nonoperative readmissions);
- Open or laparoscopic reoperations that could potentially be complicated by present adhesions.

If the patient chart had no record of adhesion-related readmission within approximately 2 years of the index surgery, the patient was requested to complete a questionnaire after obtaining written consent. The questionnaire was also essential if a patient chart had a record of adhesion-related readmission within approximately 2 years of the index surgery and further information or clarification was needed. The questionnaire collected information regarding readmission or reoperation that was directly or possibly related to adhesions following the index gynecologic surgical procedure at a hospital or outpatient clinic other than the study sites where the initial surgery was performed.

The study was approved by the institutional review board (IRB) and the independent ethics committee (IEC) in each country. Firstly, the ethic approval was granted by the Medical Ethics Committee of the Carl von Ossietzky University Oldenburg (approval code 2023-107, dated 22 August 2023). Secondly, approval was granted by the Western-Copernicus Group IRB (approval code 20231728, dated 28 November 2023). A waiver of informed consent was granted by the ethic boards at the participating sites for the chart review. The investigators obtained valid consent from each patient who completed the questionnaire for the prospective data collection related to this study, as required by the ethic boards at the participating centers. The study was conducted in accordance with Good Clinical Practice guidelines and the Declaration of Helsinki. Participating investigational sites were responsible for complying with applicable regional or national regulations governing the conduct of post-market surveillance (follow-up) studies. This trial was registered on ClinicalTrials.gov (NCT05811585).

The primary analysis examined the incidence of hospital readmissions within 2 years from the date of index surgery that were directly or possibly related to adhesions (primary endpoint) using the data provided in the chart review. The study included only adult female patients who had undergone laparoscopic gynecological surgery two years prior and had received 4% icodextrin instillation to reduce postoperative adhesions.

Patients were assumed event-free if no adhesion-related events were identified in the chart. Additionally, a subgroup analysis of the primary endpoint was carried out to determine the effect of prior surgeries on the incidence of reoperations or readmissions.

The supplemental analysis investigated the incidence using data from both the patient chart and the questionnaire. Patients were considered to have adhesion-related readmissions or reoperations if it was identified from either the chart review or patient-reported questionnaire. For the chart review portion of the supplemental analysis, patients were only included for analysis if there was evidence of 2-year follow-up in the chart. The questionnaire was intended to fill the evidence gap in cases where patients were treated for adhesion-related complications in other health systems within a 2-year window. Therefore, patients included in the supplementary analysis are those who responded to the questionnaire, who had a record of reoperation or readmission in the chart, and/or who had follow-up reported within 2 years of index surgery, as shown in the patient disposition flow chart.

The safety endpoint was assessed by collecting adverse events (AEs), serious adverse events (SAEs), and any other AEs potentially related to the device or procedure, as recorded in patient charts. The collection period was defined as the date of the index surgery to 2 years post operation.

Data were summarized using descriptive statistics for all eligible patients. Continuous variables were summarized by sample size (*n*), mean, standard deviation, median, minimum, and maximum. Frequency and percentages were provided for categorical variables. The exact 95% binomial confidence interval (CI; Clopper–Pearson) was provided for the incidence rate of adhesion-related events. Data from the patient questionnaire were summarized using descriptive statistics, and missing values were not imputed.

## 3. Results

**Figure 1:**
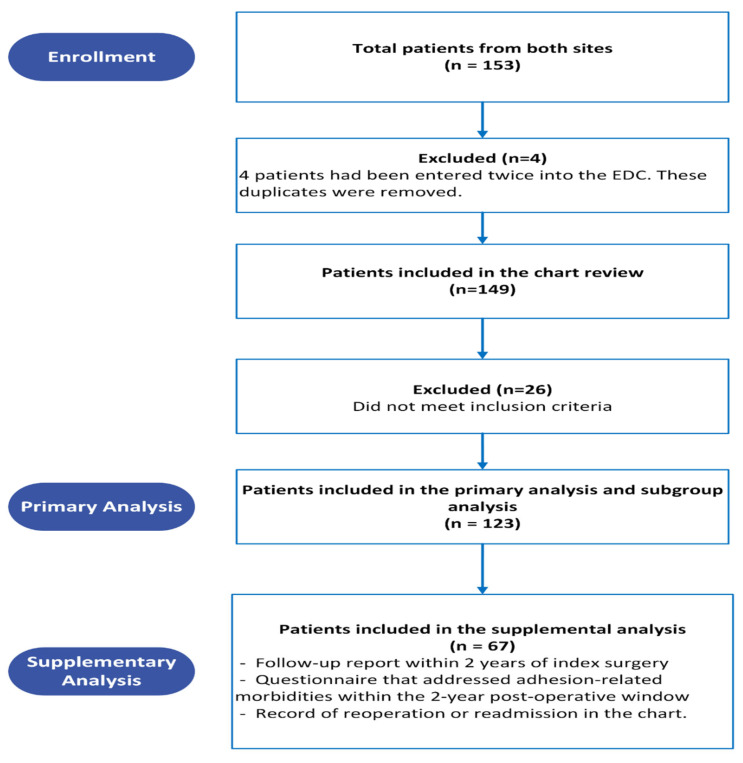
Patient disposition.

A total of 153 charts from both study sites were screened; 149 patients were included in the chart review (Figure 1), and 26 patients were excluded due to meeting one or more of the exclusion criteria, or because they returned invalid questionnaires.

A supplemental analysis was performed on 67 patients using data from both the chart and the questionnaire (Table 1).

### Demographics and Baseline Characteristics

As shown in Table 2, the mean age of the 123 patients included in the primary analysis was 37.6 years (SD ± 11.50). Of these, 98 (79.7%) patients had prior abdominal/pelvic surgeries, whilst 25 (20.3%) did not have any prior abdominal/pelvic surgeries at the moment of the index surgery. The mean height (cm), weight (kg), and BMI (kg/m^2^) were 166.32 (SD ± 7.212, *n* = 120), 73.05 (SD ± 17.395, *n* = 121), and 26.28 (SD ± 5.666, *n* = 120), respectively.

Notably, of the 123 patients, there were 68 (55.3%) patients who were reported having endometriosis and 46 (37.4%) patients who were reported to have pelvic adhesions in their medical history (Table 3).

The reported diagnosis at index procedure is shown in Table 4. From the 123 patients included, 66 (53.7%) had a diagnosis of endometriosis, 43 (35.0%) had a diagnosis of ovarian cysts, 2 (1.6%) had a diagnosis of malignancy, 21 (17.1%) had a diagnosis of fibroids/myoma, and 110 (89.4%) were classified as “other”. Of these “other” diagnoses, 105 were adhesion-related. A total of 122 patients had procedures performed, 108 (87.8%) had lysis of adhesions, 65 (52.8%) had endometriosis surgery, 63 (51.2%) had diagnostic laparoscopy, 31 (25.2%) had ovarian cystectomy, 20 (16.3%) had myomectomy, 16 (13.0%) had ovarian resection, 1 (0.8%) had a hysterectomy, and 17 (13.8%) had procedures which were classified as “other”.

**Results of study endpoints**

Primary analysis

The primary analysis (Table 5), which considered only reoperations or readmissions in the chart review, showed that 4 out of the 123 patients (3.3%) (95% CI: 0.89% to 8.12%) had at least one reoperation or readmission that was directly or possibly related to adhesion within 2 years of index surgery in their charts.

Overall, 15 reoperations or readmissions were identified in the patient charts (Table 6), 10 of which were within the 2 years from index surgery, while 5 were more than 2 years from index surgery. For those 10 events, 2 were assessed by the investigators to be directly related to adhesions (2 patients), and 5 were possibly related to adhesions (2 patients).

**Supplemental Analysis Results**

A supplemental analysis was conducted using the data from both the chart review and the questionnaire. Of the 123 patients included in the primary analysis, 67 patients were included in the supplemental analysis. Of these 67 patients, 59 patients responded to the questionnaire, 4 patients did not respond to the questionnaire but had a record of reoperation or readmission in the chart, and another 4 patients did not respond to the questionnaire and had no reoperation or readmission in the chart but had documented evidence in the chart for at least 2 years of follow-up.

Of these 67 patients included in the supplemental analysis, 5 patients reported a readmission or reoperation that was directly or possibly related to adhesions within 2 years of index surgery in their questionnaire, with 2 being reported in both the chart review and questionnaire (Table 7). For the supplemental analysis calculation, these were each counted once. Thus, 7 of 67 patients were identified as having reoperations or readmissions within 2 years that were directly or possible related to adhesions, as recorded on either patient questionnaires or in the chart review, which resulted in an incidence rate of 10.4% (95% CI 4.30% to 20.35%).

A worst case imputation sensitivity analysis was also performed, including the three reoperations/readmissions with missing data from the questionnaires for a total of 10 patients, which translates to a 14.9% (95% CI: 7.40% to 25.74%) incidence rate (Table 8). The reasons for readmission, as indicated in the questionnaire, were endometriosis stage 4, adhesions, or pelvic pain. The events were classified as potentially related to adhesions.

**Subgroup analysis of the primary endpoint, stratified by prior surgery**

A total of 25 patients were reported to be surgically naïve, whilst 98 patients had reported prior surgeries. The results from the subgroup analysis showed an incidence rate of reoperations or readmissions of 4.0% (95% CI: 0.10% to 20.35%), as reported in the chart review, and 8.0% (95% CI: 0.98% to 26.03%) as reported in the chart review or questionnaire in the surgically naïve group (N = 25). For those patients who had prior surgery (N = 98), these values were 3.1% (95% CI: 0.64% to 8.69%) and 5.1% (95% CI: 1.68% to 11.51%), respectively.

An additional worst case imputation sensitivity analysis was performed including the three patients with incomplete information in the questionnaire. The incidence rates for this analysis were 8.0% (95% CI: 0.98% to 26.03%) for the surgically naïve group and 8.2% (95% CI: 3.59% to 15.45%) for the group who had prior surgery (Table 9).

**Safety Analysis**

There were no adverse events related to 4% icodextrin reported in this study.

## 4. Discussion

This retrospective study was a single-arm, two-center review with a patient questionnaire to supplement the patient chart, with the aim to substantiate if 4% icodextrin reduces post-surgical adhesions in patients undergoing gynecological laparoscopic surgery. There were no safety concerns, nor any new safety signals identified. The data collected in this study indicate that 4% icodextrin is safe and effective as an intraperitoneal instillate in gynecological laparoscopic procedures.

The most obvious and effective strategy for adhesion prevention is to avoid surgery. If this is not an option, surgical techniques that minimize trauma and postoperative contact of injured sites should be chosen. For this purpose, careful tissue handling and minimization of electrocoagulation should be implemented, and manipulation of the peritoneum should be avoided as much as possible. Care should be taken to avoid chronic inflammatory reactions that may be caused by foreign bodies, and to support adhesion formation. Since laparoscopic approaches produce less peritoneal trauma than open surgery, adhesion formation can indeed be reduced but not prevented. Even laparoscopy introduces new problems like hypoxia of peritoneal cells, which can be induced by increased intra-abdominal pressure compressing supporting blood vessels or through laparoscopic graspers that apply far more pressure on tissues than what is actually needed to hold them, thereby causing significant tissue damage. Furthermore, the level of desiccation and abrasion and the subsequent damage to peritoneal cells can be minimized through frequent moistening of the surgical area, as well as through the gas used and by avoiding dry swabs and towels and reducing heat and light. An epidemiological study found that the overall risks of adhesion-related readmission following laparoscopic and open surgeries are comparable [8]. Postoperative adhesions are common in almost all surgical fields and are associated with morbidity, mortality, and a significant decrease in quality of life. They can lead to intestinal obstruction, chronic abdominopelvic pain, secondary subfertility, organ dysfunction, and increased requirement of and difficulty in following surgeries, such as surgical lysing of adhesions [9], which significantly reduces the life quality of patients. Adhesions in patients, particularly women, may warrant further surgeries, which are often more complex and can lead to additional complications [10]. No single approach has been satisfactory in reducing adhesions [11]. Advances in surgical techniques, such as laparoscopic surgery, can help minimize the probability of adhesion formation. No product has shown to be a substitute for good surgical technique, and the best approach to adhesion reduction may be a combination of treatments that are designed to minimize the adhesion forming process from the outset [12], based on the inevitable peritoneal injury inflicted by any type of surgery. As a consequence, there continues to be a need for anti-adhesion barriers and agents in open and laparoscopic surgery. The ideal barrier agent should be biodegradable, laparoscopically applicable, clinically efficacious, and affordable for daily routine use.

The results from our study in patients treated with 4% icodextrin show an overall readmission rate of 3.3% within 2 years when factoring in readmissions/reoperations which were either directly or possibly related adhesion. It should be noted that the patient population was deemed to be at high risk for adhesion-related complications, with the most common index diagnosis being endometriosis and/or pelvic adhesion and the most common index procedure being adhesiolysis and endometriosis resection.

4% icodextrin is not considered a breakthrough product, as it has been well-established for more than 20 years and is the only approved hydroflotation device for reducing postoperative adhesions after gynecological laparoscopy. It acts to separate injured serosal surfaces during the period of peritoneal healing through the hydroflotation principle. Studies have also shown that 4% icodextrin solution is safe [13,14], well-tolerated [15], effective [13], and easy to use [15] when used in laparoscopic gynecologic surgeries. However, there was still a gap in the clinical literature, which this study filled. Lower et al. (SCAR Study, 2004) [8] reported that at 2 years, readmissions related to adhesions in medium- and high-risk laparoscopic gynecological patients without the use of any type of adhesion barrier was 2.1% (directly related) and 15.1% (possibly related). A Cochrane database review by Ahmad et al. [16] concluded that gels and hydroflotation agents appear to be effective adhesion prevention agents for use during gynecologic surgery. Participants were less likely to have adhesions at second-look laparoscopies when they received a hydroflotation agent or a gel than when they received no treatment. There is no evidence that these agents improve fertility outcomes or pelvic pain, because these endpoints were not assessed in the study. However, the study recommends further research to confirm the success rates in this field of study [16]. A more recent retrospective study by Krielen et al., published in 2020 [17] using these SCAR data, has shown readmissions that are directly related to laparoscopic surgery within 2 years to be approximately 8%, and approximately 11% for cases that are possibly related. The overall readmissions and reoperations reported in the present study are lower than those reported in these data, despite this study population being predominantly high-risk considering their medical history, because of the high rate of adhesiogenic endometriosis and of pre-existing adhesions.

This study presents an original analysis of the clinical impact on hospital readmission and reoperation rates in patients undergoing high-risk gynecological laparoscopic surgery due to the use of 4% Icodextrin as an adhesion prophylactic agent. Notwithstanding, the study had limitations in that it was a retrospective analysis without a control group, whose patient population was high-risk (endometriosis or prior adhesions). The supplemental analysis may have a potentially biased population by only including those patients with identified events and confirmed 2-year follow-up after having completed the questionnaire.

## 5. Conclusions

A multitude of barriers are available for adhesion prevention, but given the multifactorial nature of adhesion development, there is no ideal agent to address such a complex pathophysiology that is not limited to the specific site of surgery. Adhesion barriers have demonstrated the potential to reduce the extent and severity of adhesion formation. Adhesion prevention agents, such as hydroflotation fluids that act all over the abdominal cavity, are not limited to a specific site. Reduction in de novo adhesion and adhesion formation on visceral sites and the abdominal wall has been observed in gynecologic procedures. 4% icodextrin has been used since 2006 and continues to be evaluated, investigated, and reported safe and efficacious in a variety of clinical settings, abdominopelvic operations, locations and patient populations. Clinical trial investigations have involved more than 5500 [8,9,11,12,13,14,15,18,19,20,21,22,23,24,25,26,27] patients worldwide. The accumulated clinical information on this instilled anti-adhesion agent thoroughly demonstrates that the product is well-tolerated and appropriate for use in patients undergoing various types of laparoscopic abdominal and pelvic surgery, showing clinical benefits. 4% icodextrinis is recognized as an easy-to-use device that is widely employed in laparoscopic gynecological surgeries for adhesion reduction. Developments in adhesion reduction strategies and new agents offer a realistic possibility of reducing the risk of adhesion formation, thus improving the outcomes for patients and the associated onward burden for patients, surgeons, and healthcare systems. Available clinical data on anti-adhesion agents show encouraging results to complement good surgical practice to prevent adhesions.

This is the first study ever on readmission and reoperation rates of a specific adhesion prophylactic agent, namely icodextrin, showing, based on the international literature, better results than in patients with a comparable gynecological surgery without application of a protecting medical product. We encourage future research on adhesion barriers to address the growing need for the effective management of postoperative adhesions while looking for a preventative strategy for patients and improving outcomes.

## Figures and Tables

**Table 1:** Patient disposition for the supplemental analysis.

Disposition Event	Total N = 149 (%)
Patients included in chart review	149 (100.0%)
Patients included in the primary analysis	123 (82.6%)
Patient’s follow-up time (days)	
*n*	122
Mean (SD)	314.9 (424.81)
Median	54.0
Min, Max	3, 1519
Total number of patients eligible for supplemental analysis	67 (54.5%)
Patients who responded to questionnaire	59 (48.0%)
Patients who did not respond to questionnaire, but had reoperation or readmission record in chart	4 (3.3%)
Patients who did not respond to questionnaire, had no reoperation or readmission in chart, but had at least 2 years of follow-up	4 (3.3%)

**Table 2:** Demographics and baseline characteristics.

Characteristics	Category	Total N = 123 *n* (%)
Age (years)	*n*	123
	Mean (SD)	37.6 (11.5)
	Median	35.0
	Min, Max	19, 80
Sex [*n* (%)]	Female	123 (100.0)
	Male	0
Race [*n* (%)]	American Indian or Alaska Native	0
	Asian	7 (5.7)
	Black or African American	7 (5.7)
	Native Hawaiian or Other Pacific Islander	0
	White	19 (15.4)
	Other	0
	Unknown and/or Not Reported	90 (73.2)
	Not Applicable	0
Prior abdominal/pelvic surgeries? [*n* (%)]	Yes	98 (79.7)
	No	25 (20.3)
BMI (kg/m^2^)	*n*	120
	Mean (SD)	26.28 (5.7)
	Median	25.30
	Min, Max	17.1, 43.8

**Table 3:** Medical history.

MedDRA Preferred Term	Total N = 123 *n* (%)
Endometriosis	68 (55.3)
Pelvic Adhesions	46 (37.4)
Uterine Polyp	32 (26.0)
Uterine Leiomyoma	26 (21.1)
Cesarean Section	19 (15.4)
Hysterectomy	18 (14.6)
Appendectomy	18 (14.6)
Delivery	17 (13.8)
Ovarian cystectomy	15 (12.2)
Oocyte harvest	13 (10.6)
Appendicitis	12 (9.8)
In vitro fertilization	11 (8.9)
Congenital uterine anomaly	9 (7.3)
Abortion spontaneous	9 (7.3)
Ovarian cyst	8 (6.5)
Adhesion	7 (5.7)

NOTE: Table represents *n* (%) of patients experiencing a medical history event. It does not reflect the number of times a patient experienced the medical history event.

**Table 4:** Index procedure.

Characteristics	Category	Total N = 123 *n* (%)
Diagnosis of index admission	Endometriosis	66 (53.7)
	Fibroids/Myoma	21 (17.1)
	Malignancy	2 (1.6)
	Ovarian cyst	43 (35.0)
	Other	110 (89.4)
What was performed at index procedure? ^1^	Endometriosis excision/ablation	65 (52.8)
	Hysterectomy	1 (0.8)
	Myomectomy	20 (16.3)
	Salpingectomy	10 (8.1)
	Ovarian cystectomy	31 (25.2)
	Ovarian resection	16 (13.0)
	Lysis of adhesions	108 (87.8)
	Diagnostic laparoscopy	63 (51.2)
	Other	17 (13.8)

NOTE: The percentages in the subcategories do not total 100%, because a single patient may have multiple diagnoses or undergo multiple procedures during their index admission. ^1^ The information regarding what was performed during the index procedure for one patient was missing.

**Table 5:** Primary endpoint: incidence of reoperation or readmission that was directly or possibly related to adhesions in the chart review.

Characteristics	Total (N = 123)
Total number of patients with at least one reoperation or readmission that was directly or possibly related to adhesion within 2 years of index surgery	4
Percentage of patients with at least one reoperation or readmission that was directly or possibly related to adhesion within 2 years of index surgery (95% CI) ^1^	3.3% (0.89% to 8.12%)

^1^ 95% CI based on Clopper–Pearson method.

**Table 6:** Reoperations or readmissions that were directly or possibly related to adhesions within 2 years of index surgery in the chart review.

Patient Age at Index Surgery (Years)	Time to Reoperation or Readmission	Relationship of Reoperation or Readmission to Adhesions	Nature of Readmission
30	1 week	Directly Related	Operative readmission—Open Surgery
55	7 weeks	Possibly Related	Operative readmission—Laparoscopic/endoscopic
45	7, 20, 24, 48 weeks	Possibly Related	Operative readmission—Laparoscopic/endoscopic
31	81 weeks	Directly Related	Operative readmission—Laparoscopic/endoscopic

**Table 7:** Reoperations or readmissions that were directly or possibly related to adhesions within 2 years of index surgery from the questionnaire.

Age at Index Surgery (Years)	Reoperation or Readmission Captured in Chart Review?	Reason/Diagnosis for the Hospital Readmission	Relationship of the Reoperation or Readmission to Adhesions
33	No	Adhesions/scarred tissue	Possibly related
36	No	Intestinal adhesiolysis, cystic adnexal findings	Possibly related
55	Yes	Adhesions in the pelvis and abdominal cavity	Possibly related
23	No	Cannot find the information	Possibly related
31	Yes	Endometriosis stage 4, adhesions, pelvic pain	Directly related

**Table 8:** Incidence of reoperation or readmission that was directly or possibly related to adhesions. Supplemental analysis: chart review and questionnaire.

Characteristics	Total (N = 67)
Total number of patients experiencing at least one reoperation or readmission that was directly or possibly related to adhesions within 2 years of index surgery, reported in either chart review or questionnaire	7
Incidence (95% CI) ^1^	10.4% (4.30% to 20.35%)
**Sensitivity analysis: worst case imputation**	
Total number of patients experiencing at least one reoperation or readmission that was directly or possibly related to adhesions within 2 years of index surgery, reported in either chart review or questionnaire ^2^	10
Incidence (95% CI) ^1^	14.9% (7.40% to 25.74%)

^1^ 95% CI based on Clopper–Pearson method. ^2^ Worst case imputation includes 3 patients whose questionnaires were missing the timing of reoperation and/or readmission or the relationship to adhesions.

**Table 9:** Subgroup analysis: Incidence of reoperation or readmission that was directly or possibly related to adhesions by surgical history.

Primary Endpoint Source	Surgically Naïve (N = 25)	Prior Surgery (N = 98)
Incidence in Chart Review (95% CI) ^1^	4.0% (0.10% to 20.35%)	3.1% (0.64% to 8.69%)
Incidence in Either Chart Review or Questionnaire (95% CI) ^1^	8.0% (0.98% to 26.03%)	5.1% (1.68% to 11.51%)
**Sensitivity analysis: worst case imputation ^2^**		
Incidence in Either Chart Review or Questionnaire (95% CI) ^1^	8.0% (0.98% to 26.03%)	8.2% (3.59% to 15.45%)

^1^ 95% CI based on Clopper–Pearson method. ^2^ Worst case imputation includes 3 patients whose questionnaires were missing the timing of reoperation and/or readmission or the relationship to adhesions.

## Data Availability

Data is unavailable due to privacy or ethical restrictions.
